# Categorical Analysis of Database Consistency in Reporting Drug–Drug Interactions for Cardiovascular Diseases

**DOI:** 10.3390/pharmaceutics16030339

**Published:** 2024-02-28

**Authors:** Liana Suciu, Sebastian Mihai Ardelean, Mihai Udrescu, Florina-Diana Goldiş, Daiana Hânda, Maria-Medana Tuică, Sabina-Oana Vasii, Lucreţia Udrescu

**Affiliations:** 1 Department II—Pharmacology, Pharmacotherapy, “Victor Babeş” University of Medicine and Pharmacy Timişoara, 300041 Timişoara, Romania; suciu.liana@umft.ro; 2 Research Center for Pharmaco-Toxicological Evaluations, “Victor Babeş” University of Medicine and Pharmacy Timişoara, 300041 Timişoara, Romania; 3 Department of Computer and Information Technology, University Politehnica of Timişoara, 300223 Timişoara, Romania; sebastian.ardelean@student.upt.ro; 4 Department I—Drug Analysis, “Victor Babeş” University of Medicine and Pharmacy Timişoara, 300041 Timişoara, Romania; diana.goldis@umft.ro (F.-D.G.); daiana.handa@umft.ro (D.H.); medana.tuica@umft.ro (M.-M.T.); sabina.vasii@umft.ro (S.-O.V.)

**Keywords:** cardiovascular drug interactions, categorical analysis, drug databases, databases agreement, Fleiss’ kappa coefficient

## Abstract

Drug–drug interactions (DDIs) can either enhance or diminish the positive or negative effects of the associated drugs. Multiple drug combinations create difficulties in identifying clinically relevant drug interactions; this is why electronic drug interaction checkers frequently report DDI results inconsistently. Our paper aims to analyze drug interactions in cardiovascular diseases by selecting drugs from pharmacotherapeutic subcategories of interest according to Level 2 of the Anatomical Therapeutic Chemical (ATC) classification system. We checked DDIs between 9316 pairs of cardiovascular drugs and 25,893 pairs of cardiovascular and other drugs. We then evaluated the overall agreement on DDI severity results between two electronic drug interaction checkers. Thus, we obtained a *fair agreement* for the DDIs between drugs in the cardiovascular category, as well as for the DDIs between drugs in the cardiovascular and other (i.e., non-cardiovascular) categories, as reflected by the Fleiss’ kappa coefficients of κ=0.3363 and κ=0.3572, respectively. The categorical analysis of agreement between ATC-defined subcategories reveals Fleiss’ kappa coefficients that indicate levels of agreement varying from *poor agreement* (κ<0) to *perfect agreement* (κ=1). The main drawback of the overall agreement assessment is that it includes DDIs between drugs in the same subcategory, a situation of therapeutic duplication seldom encountered in clinical practice. Our main conclusion is that the categorical analysis of the agreement on DDI is more insightful than the overall approach, as it allows a more thorough investigation of the disparities between DDI databases and better exposes the factors that influence the different responses of electronic drug interaction checkers. Using categorical analysis avoids potential inaccuracies caused by particularizing the results of an overall statistical analysis in a heterogeneous dataset.

## 1. Introduction

A drug–drug interaction (DDI) occurs between two drugs when both are simultaneously in a biological system, and one drug influences the activity of the other, pharmacokinetically or pharmacodynamically. DDIs may increase or decrease the beneficial or adverse effects of the associated drugs. Thus, the biggest concern is that DDIs may alter the expected effects or boost the adverse effects of drugs, resulting in a high percentage of hospitalizations [1,2]. Such problems are frequent, as many clinical circumstances require the combination of multiple drugs. In this context, polypharmacy—the association of at least five drugs [3,4]—emerges as a high DDI risk factor [1,2,5,6]. Elderly patients often experience polypharmacy due to comorbidities requiring complex pharmacotherapeutic regime schemes; therefore, elderly patients are prone to the risk of DDI adverse effects [7,8]. When many drugs are associated, another significant problem is healthcare professionals’ poor and inconsistent identification of drug interactions, as they frequently encounter discrepant results in the electronic drug interaction checkers (EDICs) [9,10,11,12].

This paper focuses on cardiovascular diseases (CVDs) because of their high prevalence and patient exposure to frequent and risky DDIs. Indeed, according to the World Health Organization, CVDs cause almost 18 million deaths/year worldwide, thus being the leading cause of death [13]. Hypertension, coronary heart disease, cerebrovascular disease, rhythm disorders, and other heart and blood vessel conditions are CVDs that affect more than 500 million people worldwide in 2023 [14,15]. Moreover, polypharmacy with severe potential DDIs is predominant in elderly patients with CVDs [16,17].

State-of-the-art literature reveals various population-based approaches to analyzing drug–drug interactions in cardiovascular diseases. One strategy is to investigate cohort studies reporting the prevalence of DDIs in specified clinical circumstances, e.g., a retrospective cross-sectional study on CVD patients admitted to the cardiology ward [18], a prospective observational study on hospitalized CVD patients at cardiology departments [19], or a retrospective survey of patients ≥ 65 years [16]; these studies searched for DDIs with the Lexicomp drug interactions screening tool records. Another cohort-based strategy is to compare the results of various EDICs for specific categories of cardiovascular drugs (e.g., statin DDIs [20]) or for DDIs between cardiovascular medicines and drugs from other classes (e.g., cardiac–psychiatric drug combinations [21]).

**Figure 1:**
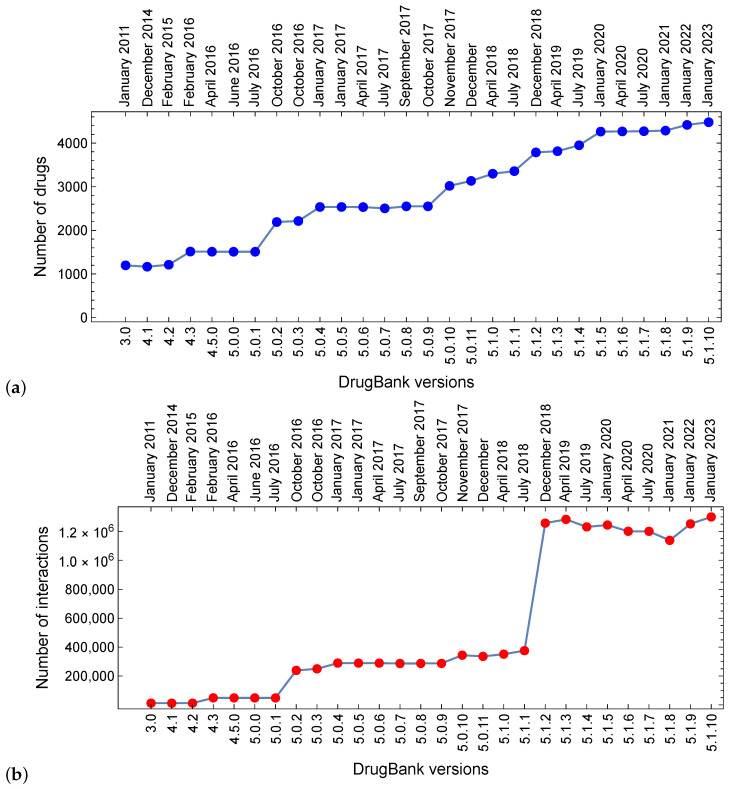
The growth in the number of drugs involved in drug–drug interactions (**panel a**) and the number of drug–drug interactions (**panel b**) over the period 2011–2023, as reflected in the evolution of DrugBank versions (3.0 to 5.1.10): We built drug–drug interaction (DDI) networks with data from successive DrugBank versions and then counted the number of drugs and their interactions in the largest DDI network components, according to the procedure presented in [35].

The literature review shows that EDICs are valuable resources in clinical decision support. However, inconsistent results are frequently reported when using such tools, and this situation tends to worsen as the number of drug–drug interactions (DDIs) and drugs involved in DDIs has increased markedly over the last five years (see Figure 1). This observation led to the use of various statistical methods to estimate the extent of agreement between drug databases that underpin EDICs.

An indicator of EDIC inconsistency is the number of drugs shared by their databases, which consequently affects the number of DDIs in each EDIC [22,23,24,25,26,27,28]. A straightforward method to evaluate the agreement between EDICs and their databases is the percentage comparison of DDIs classified on different levels of severity [12,23,24,25,26,27,29,30]. A more elaborated method is calculating kappa and weighted kappa Fleiss’ coefficients as quantitative measures of agreement between online drug interaction checkers [11,22,28,31,32]. Some studies have combined the two methods [9,10,33], and others have used the Jaccard similarity coefficient to assess global agreement between drug databases [20]. The literature also discloses wide variations in the number of checked DDIs and EDICs: 15 drug pairs checked using 3 EDICs [34], 100 and 125 drug pairs tested using 6 EDICs [10,33], 1393 and 1382 DDIs checked in the 2021 and 2022 successive versions of 4 EDICs [11], and around 2500 DDIs generated by 182 drugs checked against 3 EDICs [28].

Our manuscript offers an original, more detailed approach by analyzing DDIs according to the cardiovascular drugs’ pharmacotherapeutic subcategories, as expressed by Level 2 of the ATC (Anatomical Therapeutic Chemical) classification system. (Previous approaches delivered mainly overall statistical analyses on the EDICs agreement.) Individually or by association, CVDs often require complex cardiovascular therapeutical regimens consisting of drugs from different level 2 ATC pharmacotherapeutic categories; therefore, we analyzed the interactions between pairs of drugs acting on the cardiovascular system. Furthermore, considering that cardiovascular diseases are frequently associated with other pathologies, we checked DDIs between cardiovascular drugs and drugs for other diseases.

Accordingly, the objectives of our study are as follows:

1. to evaluate the incidence, types, and severity of drug–drug interactions (DDIs) of cardiovascular drug subcategories according to the Anatomical Therapeutic Chemical (ATC) classification system;
2. to assess the agreement on cardiovascular DDI severity results between two EDICs (Drugs.com and WebMD.com) with Fleiss’ kappa coefficient.

In achieving the stated objectives, the main original contribution of our study is to show that the overall EDIC agreement metrics, calculated on the entire dataset, can be misleading. (For instance, an overall *fair agreement* may not apply to drug subcategories; we may find even *perfect agreement* or *poor agreement* of DDI severity in Level 2 ATC subcategories.) We also discuss why we find such variation in DDI severity agreement for drugs in distinct cardiovascular subcategories. Overarchingly, our study’s findings have helpful implications for the way that healthcare professionals use and rely on EDICs in clinical practice.

## 2. Materials and Methods

We describe the statistical method we used to assess the agreement of the drug dataset following the objectives we stated in Section 1; then, we explain how we gather the data required for such an analysis.

### 2.1. Problem Statement

To calculate the level of agreement in reporting DDI severity, we used the established Fleiss’ kappa [36], a metric for inter-rater agreement, where the response variable is the DDI severity level (i.e., a categorial scale measurement), and the raters are the distinct datasets we consider in our study (i.e., Drugs.com [37] and WebMD.com [38]. The Fleiss’ kappa statistical metric is given as

(1) $$\kappa =\frac{\bar{P}-{\bar{P}}_{e}}{1-{\bar{P}}_{e}},$$

where

(2) $$\bar{P}=\frac{1}{N}\sum_{i=1}^{N}P_{i}=\frac{1}{nN(n-1)}\left(\sum_{i=1}^{N}\sum_{j=0}^{l-1}n_{ij}^{2}-nN\right),$$

and

(3) $$\bar{P_{e}}=\sum_{j=0}^{l-1}p_{j}^{2}=\frac{1}{n^{2}N^{2}}\sum_{j=0}^{l-1}{\left(\sum_{i=1}^{N}n_{ij}\right)}^{2}.$$

In Equations (2) and (3), $P_{i}$ is the extent to which the databases agree on the *i*-th DDI, $p_{j}$ is the proportion of all assignments/classifications to the *j*-th DDI severity level, *N* is the number of DDIs (i∈{1,2,…,N}), *l* is the number of severity levels (in our case, *l* = 5 and j∈{0,1,2,3,4}), *n* is the number of raters of DDIs (i.e., drug databases; *n* = 2, corresponding to Drugs.com [37] and WebMD.com [38], and $n_{ij}$ is the number of raters (i.e., datasets) that assign the *i*-th interaction to the severity level *j*.

As our study focuses on cardiovascular drug–drug interactions, we have two drug types: cardiovascular (*C*) and other (non-cardiovascular) that interact with cardiovascular drugs (*O*). Accordingly, |C| drugs are in the cardiovascular category and |O| drugs in the others category; therefore, |C|+|O|=N. (|S| represents the number of elements in set *S*.)

Pairs of cardiovascular drugs in category *C* can also generate DDIs; consequently, we perform two types of analyses: (1) between drugs in category *C* and (2) between drugs in category *C* and drugs in category *O*. Accordingly, we instantiate the $\bar{P}$ and ${\bar{P}}_{e}$ expressions in Equations (2) and (3):

(1) DDIs between drugs in category *C*: we instantiate $\bar{P}$ as
  (4) $${\bar{P}}^{C}=\frac{1}{\left|C|(|C|-1\right)}\sum_{i=1}^{\left|C|(|C|-1\right)}P_{i}.$$
  Therefore, in the case of DDIs between drugs in category *C*, Equation (2) becomes
  (5) $${\bar{P}}^{C}=\frac{1}{n(n-1)|C|(|C|-1)}\left[\sum_{\begin{array}{c} u,v\in C\\ u\neq v \end{array}}\sum_{j=0}^{l-1}{\left(n_{uv}^{j}\right)}^{2}-{n(|C|}^{2}-\left|C|\right)\right].$$
  Using the same logic, we obtain the instantiation of ${\bar{P}}_{e}$ from Equation (3) as
  (6) $${\bar{P}}_{e}^{C}=\sum_{j=0}^{l-1}p_{j}^{2}=\frac{1}{n^{2}{\left|C\right|}^{2}{\left(|C|-1\right)}^{2}}\sum_{j=0}^{l-1}\sum_{j=0}^{l-1}{\left(n_{uv}^{j}\right)}^{2}-{n(|C|}^{2}-|C|).$$
  In Equations (5) and (6), $n_{uv}^{j}$ is the number of databases that assign severity *j* to the DDI between drugs u,v∈C, u≠v.
(2) DDIs between drugs in category *C* and drugs in category *O*: in the case of DDIs between drugs in category *C* and drugs in category *O*, Equation (2) becomes
  (7) $${\bar{P}}^{O}=\frac{1}{\left|C|\cdot |O\right|}\sum_{i=1}^{\left|C|\cdot |O\right|}P_{i}=\frac{1}{n(n-1)|C|\cdot |O|}\left[\sum_{\begin{array}{c} u\in C\\ v\in O \end{array}}\sum_{j=0}^{l-1}{\left(n_{uv}^{j}\right)}^{2}-\left|C\right|\cdot \left|O\right|\right].$$
  The instantiation of ${\bar{P}}_{e}$ from Equation (3) is
  (8) $${\bar{P}}_{e}^{O}=\sum_{j=0}^{l-1}{p_{j}}^{2}=\frac{1}{n^{2}{\left|C\right|}^{2}{\left|O\right|}^{2}}\sum_{j=0}^{l-1}{\left(\sum_{\begin{array}{c} u\in C\\ v\in O \end{array}}n_{uv}^{j}\right)}^{2}$$
  In Equation (7) and (8), $n_{uv}^{j}$ is the number of databases that assign DDI severity *j* to the DDI between drug u∈C and drug v∈O.

### 2.2. Preparing Data for the EDIC Agreement Test

According to our objectives, we consider DDIs among cardiovascular drugs and DDIs between cardiovascular drugs and other most frequently associated drugs. To this end, we used DrugBank’s ATC classification list [39] to select drug subcategories corresponding to ATC Level 2 included in the Cardiovascular System category *C*. We included in *C* all approved active substances listed by DrugBank in subcategories $C_{01}$—*Cardiac therapy*, $C_{02}$—*Antihypertensives*, $C_{03}$—*Diuretics*, $C_{04}$—*Peripheral vasodilators*, $C_{07}$—*Beta blocking agents*, $C_{08}$—*Calcium channel blockers*, $C_{09}$—*Agents acting on the renin-angiotensin system*, and $C_{10}$—*Lipid modifying agents*. Using the formalism from Section 2.1, the set of cardiovascular drugs we consider is the union of the mentioned subcategory sets, $C=C_{01}\cup C_{02}\cup C_{03}\cup C_{04}\cup C_{07}\cup C_{08}\cup C_{09}\cup C_{10}$.

We then extracted drugs in *O* as non-cardiovascular approved active substances, which interact with drugs in *C*, listed by DrugBank in the Level 2 ATC subcategories: $A_{02}$—*Drugs for acid-related disorders*, $A_{10}$—*Drugs used in diabetes*, $A_{12}$—*Mineral supplements*, $B_{01}$—*Antithrombotic agents*, $G_{04}$—*Urologicals*, $M_{01}$—*Antiinflammatory and antirheumatic products*, $M_{04}$—*Antigout preparations*, $N_{05}$—*Psycholeptics*, and $N_{06}$—*Psychoanaleptics*. To account for the time constraints of evaluating a massive number of DDIs, we trimmed some subcategories: we selected only potassium salts from $A_{12}$—*Mineral supplements*, and drugs used in the benign prostatic hypertrophy from $G_{04}$—*Urologicals*. Drugs in $N_{05}$—*Psycholeptics* include antipsychotics, hypnotics, sedatives, and anxiolytics; however, in clinical practice, antipsychotics are seldom prescribed with cardiovascular drugs in *C*. Therefore, we decided to better capture the profile of cardiovascular drug interactions by splitting the $N_{05}$ subcategory into subsubcategories $N_{05}$-1—Antipsychotics and $N_{05}$-2—Hypnotics, sedatives, and anxiolytics. By the same logic, we split $N_{06}$ into $N_{06}$-1—Antidepressants and $N_{06}$-2—Nootropics and anti-dementia. The category of other drugs *O* is the union of mentioned non-cardiovascular drug subcategories, $O=A_{02}\cup A_{10}\cup A_{12}\cup B_{01}\cup G_{04}\cup M_{01}\cup M_{04}\cup N_{05}\text{-}1\cup N_{05}\text{-}2\cup N_{06}\text{-}1\cup N_{06}\text{-}2$.

In the subsequent step, we inspected each drug on Drugs.com [37] and WebMD.com [38] to build a list of drugs listed in both databases. Thus, from the 205 cardiovascular drugs *C* in DrugBank, we found |C|=137 drugs listed in both drug databases. From 267 drugs from other categories *O* in DrugBank, we found only |O|=189 drugs listed in both drug databases.

For the overall agreement between EDICs for drugs in *C*, and between drugs in *C* and drugs in *O*, we have a list of $MM1\;/\;2\cdot \left|C\right|\left(|C|-1\right)=9316$ pairs of cardiovascular drugs, and a list of |C|·|O| = 25,893 pairs of cardiovascular-other drugs, respectively; for each such pair, we manually checked its DDI severity on Drugs.com [37] and WebMD.com [38].

Equation (4)–(8) correspond to the overall agreement assessment. For the agreement analysis on drug subcategories, we use the subcategories we defined for both *C* and *O*; therefore, we form pairs only between drugs from distinct subcategories. Accordingly, we gathered DDI severity values, recorded in the considered EDICs, for pairs of drugs from the same cardiovascular category *C* but different subcategories (i.e., one drug is in subcategory $C_{x}$, and the other in subcategory $C_{y}$, with x≠y, $x,y\in \left\{01,02,03,04,07,08,09,10\right\}$). Altogether, the analysis performed on *C* subcategories entails $\sum_{\begin{array}{c} x,y\\ x<y \end{array}}|C_{x}\left|\cdot \right|C_{y}|=7658$ drug pairs. (The numbers of drugs in each *C* subcategory are $|C_{01}\left|=46,\right|C_{02}|=11$, $|C_{03}\left|=15,\right|C_{04}\left|=4,\right|C_{07}\left|=13,\right|C_{08}\left|=11,\right|C_{09}\left|=19,\right|C_{10}|=18$).

The agreement analysis for DDIs between drugs in category $C_{x}$ and drugs in $C_{y}$ requires the instantiation of $\bar{P}$ as

(9) $${\bar{P}}_{xy}^{C}=\frac{1}{|C_{x}\left|\cdot \right|C_{y}|}\sum_{i=1}^{|C_{x}\left|\cdot \right|C_{y}|}P_{i}=\frac{1}{n\left(n-1\right)|C_{x}\left|\cdot \right|C_{y}|}\left[\sum_{\begin{array}{c} u\in C_{x}\\ v\in C_{y} \end{array}}\sum_{j=0}^{l-1}{\left(n_{uv}^{j}\right)}^{2}-|C_{x}\left|\cdot \right|C_{y}|\right],$$

and of ${\bar{P}}_{e}$ as

(10) $${\bar{P}}_{e}^{C_{x}C_{y}}=\frac{1}{n^{2}|C_{x}{|}^{2}{\left|C_{y}\right|}^{2}}\sum_{j=0}^{l-1}{\left(\sum_{\begin{array}{c} u\in C_{x}\\ v\in C_{y} \end{array}}n_{uv}^{j}\right)}^{2},$$

where $n_{uv}^{j}$ is the number of databases that assign DDI severity *j* to the DDI between drug $u\in C_{x}$ and drug $v\in C_{y}$.

For the agreement analysis between drugs in *C* subcategories and drugs in *O* subcategories, we kept the DDI severity values recorded in the two EDICs we consider for pairs of drugs where one drug is in subcategory $C_{x}$, and the other in subcategory $O_{y}$ ($x\in \left\{01,02,03,04,07,08,09,10\right\}$, $y\in \left\{01,02,03,04,05,06,07,08,09,10,11\right\}$, with $O_{01}=A_{02}$, $O_{02}=A_{10}$, $O_{03}=A_{12}$, $O_{04}=B_{01}$, $O_{05}=G_{04}$, $O_{06}=M_{01}$, $O_{07}=M_{04}$, $O_{08}=N_{05}\text{-}1$, $O_{09}=N_{05}\text{-}2$, $O_{10}=N_{06}\text{-}1$, $O_{11}=N_{06}\text{-}2$).

Taken together, the analysis performed on DDIs between *C* and *O* subcategories entails |C|·|O| = 25,893 pairs of cardiovascular-other drugs. (The numbers of drugs in each *O* subcategory are $|O_{01}\left|=\right|A_{02}\left|=11,\right|O_{02}\left|=\right|A_{10}\left|=23,\right|O_{03}\left|=\right|A_{12}\left|=2,\right|O_{04}\left|=\right|B_{01}\left|=36,\right|O_{05}\left|=\right|G_{04}\left|=7,\right|O_{06}\left|=\right|M_{01}\left|=19,\right|O_{07}\left|=\right|M_{04}\left|=6,\right|O_{08}\left|=\right|N_{05}\text{-}1|=29,|O_{09}\left|=\right|N_{05}\text{-}2|=20,|O_{10}\left|=\right|N_{06}\text{-}1|=31,|O_{11}\left|=\right|N_{06}\text{-}2|=5$).

Also, the agreement analysis of DDIs between drugs in subcategory $C_{x}$ and drugs in $O_{y}$ demands the instantiation of $\bar{P}$ as

(11) $${\bar{P}}^{C_{x}O_{y}}=\frac{1}{|C_{x}\left|\cdot \right|O_{y}|}\sum_{i=1}^{|C_{x}\left|\cdot \right|O_{y}|}P_{i}=\frac{1}{n\left(n-1\right)|C_{x}\left|\cdot \right|O_{y}|}\left[\sum_{\begin{array}{c} u\in C_{x}\\ v\in O_{y} \end{array}}\sum_{j=0}^{l-1}{\left(n_{uv}^{j}\right)}^{2}-|C_{x}\left|\cdot \right|O_{y}|\right],$$

and of ${\bar{P}}_{e}$ as

(12) $${\bar{P}}_{e}^{C_{x}O_{y}}=\frac{1}{n^{2}|C_{x}{|}^{2}{\left|O_{y}\right|}^{2}}\sum_{j=0}^{l-1}{\left(\sum_{\begin{array}{c} u\in C_{x}\\ v\in O_{y} \end{array}}n_{uv}^{j}\right)}^{2},$$

where $n_{uv}^{j}$ is the number of databases that assign DDI severity *j* to the DDI between drug $u\in C_{x}$ and drug $v\in O_{y}$.

### 2.3. Unifying the Characterization of DDI Severity

Table 1 presents the levels of DDI severity classification in the two drug databases. WebMD.com [38] classifies DDIs starting from *0 Interaction Found* to *Don’t Use Together*, and Drugs.com [37] divides DDIs from *Unknown* to *Major*.

After reviewing these two drug databases, one can notice that they provide apparent different levels of DDI ranking. Therefore, we examined the Drugs.com [37] recommendations provided in their *Drug Interaction Report*, Professional section, and noted that it delivers distinct recommendations for the *Major* severity level, which includes *Generally avoid*, *Additional contraception recommended*, *Adjust dose*, *Adjust dosing interval*, *Monitor closely*, and *Contraindicated*. We select the *Contraindicated* recommendation and consider it equivalent to WebMD.com’s [38] *Don’t use together* recommendation, as presented in Table 1. We then allocated to each DDI severity level a score corresponding to the interaction strength, as presented in the rightmost column of Table 1: 0 for an unknown interaction, 1 for a minor interaction, 2 for a moderate interaction, 3 for a severe interaction, and 4 for a contraindicated association of two drugs.

## 3. Results

In this section, we first present an overview of the DDI agreement percentages between drug in all categories. We then characterize the overall and categorical DDI agreement with Fleiss’ kappa.

### 3.1. Overall DDI Percentage Analysis

We stratified the agreement between the two drug databases by measuring the difference in the interaction strength code returned by each drug database for a drug pair: Agreement—the severity levels indicated by each database coincide, Mild agreement—the difference is 1 (e.g., Drugs.com [37] result is 3, and WebMD.com result is 2), Mild disagreement—the difference is 2, Disagreement—the difference is 3, and Strong disagreement—the difference is 4.

For DDIs included in the Agreement category, we also checked the distribution of levels of agreement (i.e., the percentage of DDIs listed by both drug databases as *Not found*, *Minor*, *Moderate*, *Major*, and *Contraindicated*); for example, if for a drug pair, Drugs.com [37] reports *Moderate* and WebMD.com [38] *Monitor closely*, we have the same interaction strength code 2, meaning the two databases agree on the *Moderate* severity level.

#### 3.1.1. Overall DDI Analysis between Cardiovascular Drugs

**Figure 2:**
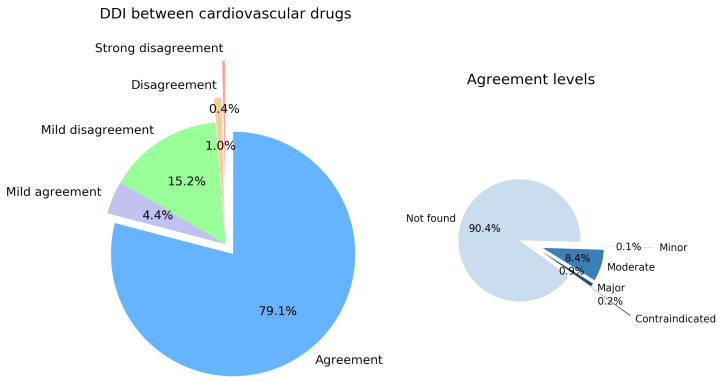
Left panel: distribution of drug–drug interactions (DDIs) between cardiovascular drugs by levels of agreement, from Agreement (if the difference between the interaction strength code is 0) to Strong disagreement (if the difference between the interaction strength code is 4). Right panel: apportionment of the DDIs database agreements according to DDIs severity levels.

Of 7658 cardiovascular DDIs analyzed using both considered EDICs, we obtained identical severity-level results for 6056 cardiovascular drug pairs, representing consensus results for 79.1% of cardiovascular DDIs between the two drug interaction checker tools. The pie chart in Figure 2 displays the distribution of agreement levels for cardiovascular drug pairs tested (left panel, *DDI between cardiovascular drugs*). Mild agreement occurs for 337 drug pairs (i.e., 4.4%), Mild disagreement for 1162 DDIs (i.e., 15.2%), Disagreement for 77 DDIs (1.0%), and Strong disagreement for 27 cardiovascular drug pairs (0.4%).

The right panel of Figure 2 shows that the EDICs concurrently indicate *Not found* interactions for 5476 drug pairs, representing 90.4% of the agreements of 6056 for DDIs between cardiovascular drugs. We also report the agreement between the EDICs on 8 *Minor* DDIs (i.e., 0.13%), 508 *Moderate* DDIs (8.4%), 52 *Major* DDIs (0.9%), and 12 *Contraindicated* DDIs (0.2%) as cardiovascular drug pairs.

#### 3.1.2. Overall DDI Analysis between Cardiovascular and Other Drugs

**Figure 3:**
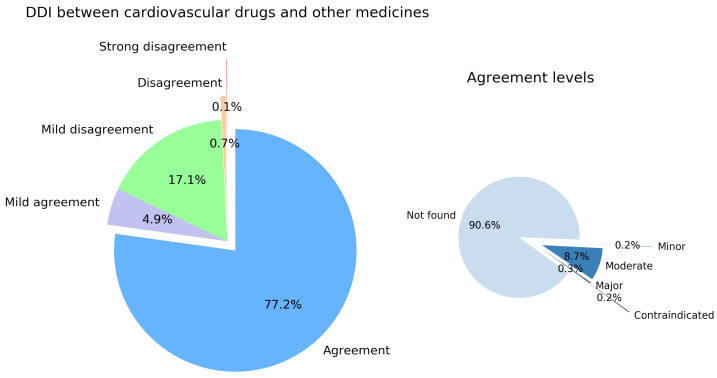
Left panel: distribution of drug–drug interactions (DDIs) between cardiovascular and other drugs by levels of agreement, from Agreement (if the difference between the interaction strength code is 0) to Strong disagreement (if the difference between the interaction strength code is 4). Right panel: the DDIs database agreements are allocated according to DDIs severity levels.

We analyzed 19,652 DDIs between cardiovascular drugs and 11 drug subcategories treating other than cardiovascular diseases. The left panel in Figure 3 indicates the allocation of the agreement categories between both EDICs. A total of 15,169 (77.2%) DDIs have the same interaction strength code, so they fall into the Agreement category. For 964 (4.9%) DDIs, we obtained a difference in the interaction strength code of 1 between the two databases; hence, they are in the Mild agreement category. Mild disagreement—represented by a difference of 2 between the corresponding interaction strength codes—occurs for 3363 (17.1%) DDIs. A total of 128 (0.7%) DDIs fit into the Disagreement category, and for 27 (0.1%) DDIs, we obtained the maximal difference of 4 between the severity levels’ codes (the Strong disagreement category).

The right panel in Figure 3 depicts the allocation of the agreements between Drugs.com [37] and WebMD.com [38] on the five severity scales. An overwhelming majority of 90.6% is represented by drug pairs for which both databases return *No interaction found*. Furthermore, the two databases agree on 32 DDIs (i.e., 0.2%) as *Minor*, 1314 DDIs (8.7%) as *Moderate*, 43 DDIs (0.3%) as *Major*, and 30 DDIs (0.2%) as *Contraindicated*.

#### 3.1.3. Categorical DDI Analysis

We also analyzed DDIs between drugs from distinct cardiovascular drug subcategories ($C_{01}$, $C_{02}$, $C_{03}$, $C_{04}$, $C_{07}$, $C_{08}$, $C_{09}$, and $C_{10}$). In the Supplementary Materials, Section S1 depicts matrices of pie charts showing the corresponding apportionment of DDIs in one of the following categories: Agreement, contraindicated; Agreement, major; Agreement, moderate; Agreement, minor; Agreement, not found; Disagreement. We provide the same analysis for DDIs between drugs in cardiovascular and other drug subcategories ($A_{02}$, $A_{01}$, $A_{12}$, $B_{01}$, $G_{04}$, $M_{01}$, $M_{04}$, $N_{05}$-1, $N_{05}$-2, $N_{06}$-1, and $N_{06}$-2) in the pie chart matrices from Section S2.

### 3.2. Drug Databases Agreement Using Fleiss’ Kappa Coefficient

We estimated the overall agreement between the two EDICs by calculating two Fleiss’ kappa coefficients corresponding to the datasets represented by cardiovascular DDIs and cardiovascular-other drug interactions, respectively.

We also calculated the kappa coefficients corresponding to each pair of ATC Level 2 subcategories of cardiovascular drugs ($C_{01}$, $C_{02}$, $C_{03}$, $C_{04}$, $C_{07}$, $C_{08}$, $C_{09}$, and $C_{10}$) and each pair of ATC Level 2 cardiovascular and other drug subcategories ($A_{02}$, $A_{01}$, $A_{12}$, $B_{01}$, $G_{04}$, $M_{01}$, $M_{04}$, $N_{05}$-1, $N_{05}$-2, $N_{06}$-1, and $N_{06}$-2), respectively.

#### 3.2.1. Fleiss’ Kappa for DDIs between Cardiovascular Drug Subcategories

We instantiate Equation (1) for drug interactions between cardiovascular type *C* drugs and obtained Fleiss’ kappa coefficient $\kappa^{C}=MM\left({\bar{P}}^{C}-{\bar{P}}_{e}^{c}\right)\;/\;\left(1-{\bar{P}}_{e}^{c}\right)=0.3572$, which indicates a *fair agreement* [40] between both EDICs.

**Figure 4:**
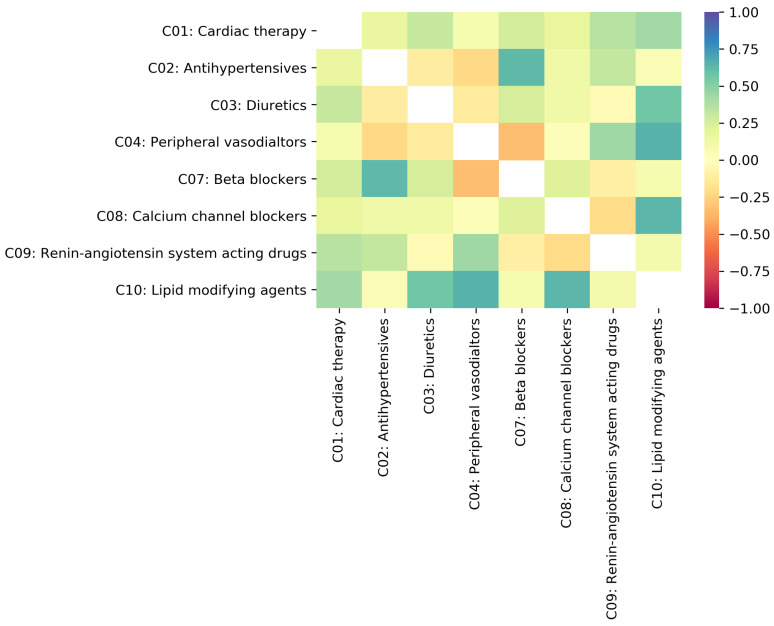
The heatmap representation of Fleiss’ kappa coefficients for drug–drug interactions between cardiovascular subcategories to assess the agreement between the EDICs we process in this paper. Color ranges from red (i.e., *poor agreement*) to dark blue (i.e., *perfect agreement*).

The two-dimensional data visualization matrix in Figure 4 illustrates the Drugs.com [37] and WebMD.com [38] κ agreement on DDIs between drugs in Level 2 cardiovascular ATC categories (from $C_{01}$ to $C_{10}$). The input rows and columns are the Level 2 ATC cardiovascular subcategories and determine the size of the matrix. The color scale ranges from red to dark blue and corresponds to a scale of κ values from −1 to 1, respectively.

Table 2 supports the heatmap in Figure 4 by presenting the Fleiss’ kappa results for the pairs of cardiovascular drug subcategories ranked according to their level of agreement; in the rightmost column, we also indicate the calculated Fleiss’ kappa interval for each level.

We obtained the highest kappa value κ=0.655 for DDIs in subcategories $C_{10}$ and $C_{04}$, which indicates *substantial agreement* between the two drug databases.

#### 3.2.2. Fleiss’ Kappa for DDIs between Cardiovascular and Other Drug Subcategories

We instantiated Equation (1) for DDIs between drugs from cardiovascular drug categories *C* and other drug types *O* and obtained Fleiss’ kappa coefficient $\kappa^{O}=MM\left({\bar{P}}^{O}-{\bar{P}}_{e}^{o}\right)\;/\;\left(1-{\bar{P}}_{e}^{o}\right)=0.3363$, which indicates a *fair agreement* [40] between the EDICS we analyze.

Table 3 presents the Fleiss’ kappa results for the pairs of drugs in cardiovascular and other drug subcategories classified based on their level of agreement between the databases we processed; in the rightmost column, we indicate the calculated Fleiss’ kappa interval for each level of agreement.

**Figure 5:**
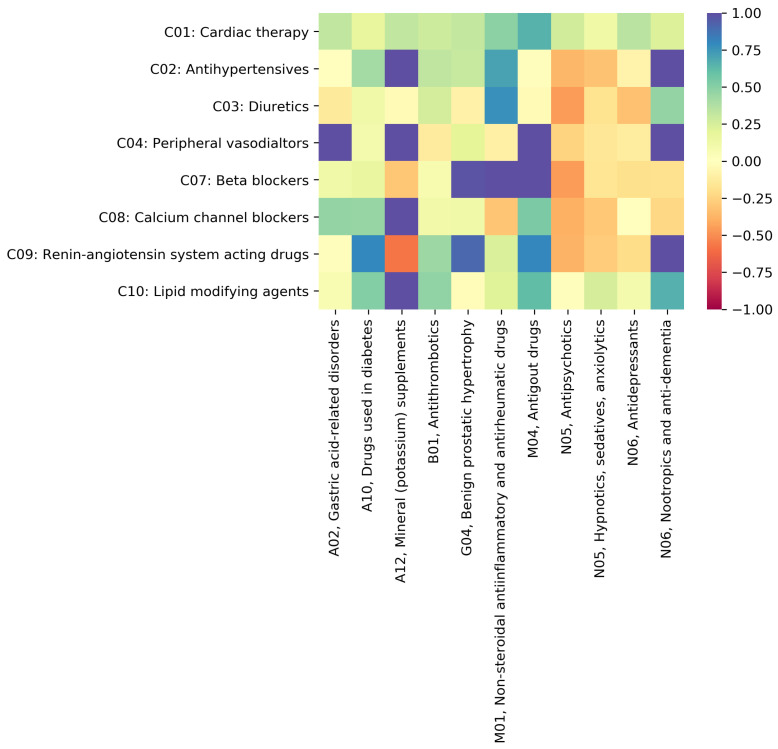
Representation of the agreement between Drugs.com [37] and WebMD.com [38] by Fleiss’ kappa coefficients for drug–drug interactions between cardiovascular and other pharmacological or therapeutic categories. Color varies from red (i.e., *poor agreement*) to dark blue (i.e., *perfect agreement*).

As presented in Table 3 and Figure 5, we have higher κ values, i.e., levels of *perfect agreement* and *almost perfect agreement* between the EDICs we examined.

## 4. Discussion

Commonly, cardiovascular patients have multidrug regimens that address cardiovascular diseases and other comorbidities. Such patients may face a significant risk of DDIs, which may cause serious health consequences. Accordingly, we compared two EDICs, namely Drugs.com [37] and WebMD.com [38], to assess the incidence, types, and severity of DDIs of cardiovascular drug subcategories (defined according to the ATC Level 2 classification system, listed in DrugBank). We noticed that the information in the two EDICs is heterogeneous, which means that the two drug databases do not include all the drugs listed by DrugBank in the investigated categories, namely *C* (i.e., cardiovascular) and *O* (i.e., other). There are also differences regarding the number of drugs included by Drugs.com [37] (i.e., 344) and WebMD.com [38] (i.e., 353); we even observed that one EDIC reports DDIs not recorded in the other EDIC. However, we note that the percentages for agreement, slight agreement, slight disagreement, disagreement, and strong disagreement between the EDICs we analyze are similar across all severity levels, which may indicate a similar pattern in the documentation and reporting of DDIs.

Indeed, the categorical analysis reveals that the two EDICs overwhelmingly agree on *Not found* DDIs between cardiovascular drugs (i.e., 79.1%) and between cardiovascular drugs and other drugs (i.e., 77.2%). As presented in the right pie charts in Figure 2 and Figure 3, the very low percentages of agreement on the *Contraindicated* and *Major* DDIs, which are the most clinically relevant interactions—in terms of severity of health effects—are noteworthy. For example, both EDICs concordantly contraindicate the combination of drug pairs such as Ivabradine ($C_{01}$)—Conivaptan ($C_{03}$), Verapamil ($C_{08}$)—Lomitapide ($C_{10}$), Dronedarone ($C_{01}$)—Sotalol, Gemfibrozil ($C_{10}$)—Repaglinide ($A_{10}$), Dronedarone ($C_{01}$)—Alfuzosin ($G_{04}$), or Mavacamten ($C_{01}$)—Ticlopidine ($B_{01}$). The two EDICs’ low concordance on the most relevant severity levels derives from the absence of a standard definition of DDI and the use of different references when documenting a specific DDI [24]. Such measures in improving the clinical relevance and evaluation of DDI evidence imply standardization in assessing and building the sets and labels of DDI severity and transferring their evidence into clinical decision support systems [35,41,42,43].

Our results also show that the highest kappa value (i.e., κ=0.655) for DDIs between cardiovascular drugs occurs for drug pairs from subcategories $C_{10}$ (Lipid modifying agents) and $C_{04}$ (Peripheral vasodilators); this indicates *substantial agreement* between the two EDICs (see Figure 4). Such a finding is consistent with the analysis of percent agreement on the DDI severity scale. Out of 72 DDIs, both EDICs agreed on *Not found* for 87.5%; there is agreement on *Major* DDIs for 5.56% and disagreement for 6.94% (see Figure S1.2 in Section S1). To illustrate the case with DDIs between drugs in $C_{10}$ and $C_{04}$, we discuss here the example of niacin ($C_{04}$) and statins ($C_{10}$). Both EDICs agree on the *Major* interaction between niacin (approved in the United States by the FDA as a prescription drug for the treatment of dyslipidemia, but not in Europe) and atorvastatin, lovastatin, pitavastatin, rosuvastatin, and simvastatin; the two EDICs are in *Mild agreement* on the interactions of niacin with fluvastatin and pravastatin. The combination of niacin—a peripheral vasodilator and the most potent drug that increases the HDL cholesterol levels [44]—and statins—very effective in lowering LDL cholesterol levels [45]—has complementary effects on the lipid profile with potential benefits in preventing cardiovascular events [46]. A 2003 review article reported rhabdomyolysis for the combination of niacin with lovastatin, pravastatin, and simvastatin; however, such interactions were not reported for the combination of niacin with atorvastatin, cerivastatin, and fluvastatin [47]. (N.B., cerivastatin was withdrawn from the market worldwide in 2001). Accordingly, clinicians should judge the advantages of niacin–statin combined therapy, given that both lipid-modifying agents have adverse effects on skeletal muscle, and closely monitor any sign of muscle disorder [46,48]. However, a more recent paper presents that niacin added to statin therapy increases multiple atherogenic HDL proteins, thus compromising the known cardioprotective effects of niacin based on raising HDL cholesterol [49]. Therefore, considering the arguments above, the current literature supports the level of *Major* severity indicated by the two EDICs; even if the pharmacokinetic differences between statins would allow the niacin association with some statins, a manufacturer of a commercial product with niacin recommends caution and careful monitoring when the doctor decides on the association. The brief literature overview in this paragraph also shows that the clinical relevance of certain DDIs is subject to volatility and uncertainty, which may reflect in *Mild agreement* and *Disagreement* between severity labels assigned by various EDICs. Nonetheless, as a rule of thumb, according to the fundamental principle of *Primum non nocere*, doctors may seriously consider the most severe DDI label from those reported in the available EDICs. Furthermore, niacin belongs to both $C_{10}$ and $C_{04}$ [39], so the niacin–statin association can be considered a therapeutic duplicate, which, in turn, may augment their adverse effects; that might explain the *Major* interactions reported by both EDICs.

The Fleiss’ kappa for cardiovascular drug categories is lowest (i.e., κ=−0.333) between subcategories $C_{07}$—*Beta blocking agents* and $C_{04}$—*Peripheral vasodilators* (see Figure 4), and indicates *poor agreement* between the two rating EDICs. Complementarily, the percentage analysis of the levels of the agreement shows that of the total of 52 DDIs, disagreement occurs for 50%; for the remaining 50%, both EDICs agree on the *Not found* label (see Figure S1.2 in Section S1). This situation is also a symptom of high uncertainty in labeling DDIs [35]; indeed, if a DDI is *Not found*, it means that it is possible that a clinically relevant DDI does not exist, but it can also mean that the DDI is yet to be uncovered.

From a clinical standpoint, our analysis strengthens the idea that drug interaction checking systems can provide valuable information but should not be the deciding factor when doctors set the pharmacotherapeutic regimen. Prescribing doctors must follow the guidelines for each pathology; however, evaluating potential DDIs becomes a complicated task when dealing with several morbidities. From a clinical standpoint, the DDI analysis using two or more EDICs is indicative to the healthcare professional as it assists in signaling potential clinically relevant DDIs with unfavorable consequences for the patient’s safety. Thus, using multiple EDICs becomes necessary in certain clinical conditions that require polypharmacy (i.e., schemes with many drugs) because the redundancy of using several EDICs can guarantee the safety of the result.

Furthermore, we also show that the clinical agreement between EDICs must compare DDIs at advanced ATC levels, i.e., three (the level of the pharmacological subgroup), four (the level of chemical/pharmacological subgroup), or more reasonably, five (the level indicating the chemical substance). Indeed, our results support this recommendation as they show that substantial agreement at ATC Level 2 is equivocal because it aggregates dissimilar results at the active substance level (ATC Level 5). Nonetheless, the main limitation of our work is using only two EDICs, and future work can correct this drawback.

The overarching conclusions of our study are as follows:

- The main agreement between DDI databases is on the *Not found* label—this indicates the lack of knowledge about many potential DDIs.
- Agreement on DDI labels other than *Not found* is rare—the general rule is that, when EDICs have information on DDIs, they tend to disagree on their severities.
- The overall assessment agreement is misleading, as it tempts to converge to a *fair agreement*, while categorical agreements present high variations.

New experiments constantly suggest new DDIs and new DDI explanations. (Figure 1, Panel b, shows the evolution of the DDI number in successive DrugBank versions). However, the DDI space is enormous and can hardly be explored even with the current experimental infrastructure; this explains the high amount of unknown DDIs, as reflected in the high percentages of *Agree, not found*. Most DDIs are complex and involve multiple factors that databases might interpret differently, leading to various levels of agreement in classifying DDIs. Furthermore, databases may use different sources or methodologies to collect data on drug interactions. Disparities in sources, inclusion criteria, or data-collecting procedures can lead to frequent inconsistent evaluations. The overall *fair agreement* result is mainly due to a large number of agreements on the *Not found* DDI label.

## 5. Conclusions

The main takeaway of this article is that the categorical analysis of the agreement on DDIs is more insightful than the overall approach, as it better exposes the differences between electronic drug interaction checking tools. The categorical analysis is objective because it avoids the potentially erroneous conclusions reached by extrapolating/generalizing the statistics of a heterogeneous and unstructured dataset or by interpolating/particularizing the results of an overall statistic analysis. Our study also reveals the drawbacks of current EDICs: a lack of standardization of criteria for DDI severity labeling and the high level of unknown DDIs.

## Figures and Tables

**Table 1:** Severity scales in the considered electronic drug interaction checkers. The first two columns display drug–drug interactions (DDIs) classification, clinical relevance, and recommendations. The third column presents the code we set for each level of DDI manifestation.

WebMD.com [38] ^1^	Drugs.com [37] ^2^ (Professional)	Interaction Strength Code
**0 Interactions Found**	**Unknown**	**0**
	No interactions were found	
**Minor**	**Minor**	**1**
Interaction is unlikely, minor, or nonsignificant		
**Monitor closely**	**Moderate**	**2**
Significant interaction possible (monitoring by your doctor required)	Monitor, Adjust dosing interval	
**Serious**	**Major**	**3**
Potential for serious interaction; regular monitoring by your doctor required or alternate medication may be needed	Generally avoid, Additional contraception recommended, Adjust dose, Adjust dosing interval, Monitor closely	
**Don’t use together**	**Major**	**4**
Never use this combination of drugs because of high risk for dangerous interaction	Contraindicated	

^1^ Severity level names are in bold font, while their corresponding WebMD.com [38] explanations are in regular font. ^2^ Severity level names in Drugs.com [37] are in bold font, while the corresponding professional explanations provided in their *Drug interaction report* are in regular font.

**Table 2:** The Fleiss’ kappa agreement between Drugs.com [37] and WebMD.com [38] for drug–drug interactions in cardiovascular drug subcategories.

Pairs of Cardiovascular Subcategories	Level of Agreement (Kappa Range)
$C_{10}$-$C_{04}$; $C_{10}$-$C_{08}$; $C_{07}$-$C_{02}$	*substantial agreement* (0.632≤ κ≤0.655)
$C_{10}$-$C_{01}$; $C_{10}$-$C_{03}$; $C_{09}$-$C_{04}$	*moderate agreement* (0.427≤ κ≤0.566)
$C_{09}$-$C_{01}$; $C_{09}$-$C_{02}$; $C_{08}$-$C_{07}$; $C_{07}$-$C_{01}$; $C_{07}$-$C_{03}$; $C_{03}$-$C_{01}$	*fair agreement* (0.221 ≤ κ≤ 0.352)
$C_{10}$-$C_{02}$; $C_{10}$-$C_{07}$; $C_{10}$-$C_{09}$; $C_{08}$-$C_{01}$; $C_{08}$-$C_{02}$; $C_{08}$-$C_{03}$; $C_{08}$-$C_{04}$; $C_{04}$-$C_{01}$; $C_{02}$-$C_{01}$	*slight agreement* (0.035 ≤ κ≤ 0.179)
$C_{09}$-$C_{03}$; $C_{09}$-$C_{07}$; $C_{09}$-$C_{08}$; $C_{07}$-$C_{04}$; $C_{04}$-$C_{02}$; $C_{04}$-$C_{03}$; $C_{03}$-$C_{02}$	*poor agreement* (κ < 0)

**Table 3:** The Fleiss’ kappa results for Drugs.com-WebMD.com agreement for drug–drug interactions between cardiovascular and other drug subcategories.

Pairs of Cardiovascular and Other Drug Subcategories	Level of Agreement (Kappa Range)
$C_{10}$-$A_{12}$; $C_{09}$-$N_{06}$-2; $C_{08}$-$A_{12}$; $C_{04}$-$M_{04}$; $C_{04}$-$A_{12}$; $C_{04}$-$N_{06}$-2; $C_{02}$-$A_{12}$; $C_{02}$-$N_{06}$-2; $C_{07}$-$M_{01}$; $C_{07}$-$M_{04}$; $C_{04}$-$A_{02}$	*perfect agreement* (κ=1)
$C_{07}$–$G_{04}$; $C_{09}$–$G_{04}$	*almost perfect agreement*
	(0.911 ≤ κ≤0.978)
$C_{09}$-$M_{04}$; $C_{09}$-$A_{10}$; $C_{03}$-$M_{01}$; $C_{02}$-$M_{01}$; $C_{10}$-$N_{06}$-2; $C_{01}$-$M_{04}$; $C_{10}$-$M_{04}$	*substantial agreement* (0.614 ≤ κ≤ 0.801)
$C_{08}$-$M_{04}$; $C_{10}$-$A_{10}$; $C_{01}$-$M_{01}$; $C_{10}$-$B_{01}$; $C_{03}$-$N_{06}$-2; $C_{08}$-$A_{02}$; $C_{08}$-$A_{10}$; $C_{09}$-$B_{01}$; $C_{02}$-$A_{10}$	*moderate agreement* (0.420 ≤ κ≤ 0.532)
$C_{10}$-$M_{01}$; $C_{10}$-$N_{05}$-2; $C_{09}$-$M_{01}$; $C_{03}$-$B_{01}$; $C_{02}$-$B_{01}$; $C_{02}$-$G_{04}$; $C_{01}$-$A_{02}$; $C_{01}$-$A_{12}$; $C_{01}$-$B_{01}$; $C_{01}$-$G_{04}$; $C_{01}$-$N_{05}A$; $C_{01}$-$N_{06}A$; $C_{01}$-$N_{06}$-2	*fair agreement* (0.212 ≤ κ≤ 0.345)
$C_{10}$-$A_{02}$; $C_{10}$-$N_{06}$-1; $C_{08}$-$B_{01}$; $C_{08}$-$G_{04}$; $C_{07}$-$A_{02}$; $C_{07}$-$A_{10}$; $C_{07}$-$A_{10}$; $C_{07}$-$B_{01}$; $C_{04}$-$A_{10}$; $C_{04}$-$G_{04}$; $C_{03}$-$A_{10}$; $C_{01}$-$A_{10}$; $C_{01}$-$N_{05}$-2	*slight agreement* (0.068 ≤ κ≤ 0.203)
$C_{10}$-$G_{04}$; $C_{10}$-$N_{05}$-1; $C_{09}$-$A_{02}$; $C_{09}$-$A_{12}$; $C_{09}$-$N_{05}$-1; $C_{09}$-$N_{05}$-2; $C_{09}$-$N_{06}$-1; $C_{08}$-$M_{01}$; $C_{08}$-$N_{05}$-1; $C_{08}$-$N_{05}$-2; $C_{08}$-$N_{06}$-1; $C_{08}$-$N_{06}$-2; $C_{07}$-$A_{12}$; $C_{07}$-$N_{05}$-1; $C_{07}$-$N_{06}$-1; $C_{07}$-$N_{05}$-2; $C_{07}$-$N_{06}$-2; $C_{04}$-$B_{01}$; $C_{04}$-$M_{01}$; $C_{04}$-$N_{05}$-1; $C_{04}$-$N_{05}$-2; $C_{04}$-$N_{06}$-1; $C_{03}$-$A_{02}$; $C_{03}$-$A_{12}$; $C_{03}$-$G_{04}$; $C_{03}$-$M_{04}$; $C_{03}$-$N_{05}$-1; $C_{03}$-$N_{05}$-2; $C_{03}$-$N_{06}$-1; $C_{02}$-$A_{02}$; $C_{02}$-$M_{04}$; $C_{02}$-$N_{05}$-1; $C_{02}$-$N_{05}$-2; $C_{02}$-$N_{06}$-1	*poor agreement* (κ < 0)

## Data Availability

The data and code supporting this paper is available at https://github.com/research-hyperion/Analysisofdatabaseconsistencyinreportingdrug-druginteractionsforcardiovasculardiseases (accessed on 10 December 2023).

## Supplementary Materials

The following supporting information can be downloaded at: https://www.mdpi.com/article/10.3390/pharmaceutics16030339/s1. Figure S1.1: The symmetric matrix of pie charts illustrating the categorical analysis for drug–drug interactions between cardiovascular drugs in subcategories $C_{01}$, $C_{02}$, $C_{03}$, and $C_{04}$; Figure S1.2: The pie charts matrix representing the categorical analysis for drug–drug interactions between cardiovascular drugs in subcategories $C_{01}$, $C_{02}$, $C_{03}$, $C_{04}$, and $C_{07}$, $C_{08}$, $C_{09}$, $C_{10}$; Figure S1.3: The symmetric matrix of pie charts illustrating the categorical analysis for drug–drug interactions between cardiovascular drugs in subcategories $C_{07}$, $C_{08}$, $C_{09}$, and $C_{04}$; Figure S2.1: The pie charts matrix representing the categorical analysis for drug–drug interactions between drugs in subcategories $C_{01}$, $C_{02}$, $C_{03}$, $C_{04}$, and $A_{02}$, $A_{10}$, $A_{12}$, $B_{01}$, $G_{04}$; Figure S2.2: The pie charts matrix representing the categorical analysis for drug–drug interactions between drugs in subcategories $C_{01}$, $C_{02}$, $C_{03}$, $C_{04}$, and $M_{01}$, $M_{04}$, $N_{05}$-1, $N_{05}$-2, $N_{06}$-1, $N_{06}$-2; Figure S2.3: The pie charts matrix representing the categorical analysis for drug–drug interactions between drugs in subcategories $C_{07}$, $C_{08}$, $C_{09}$, $C_{10}$, and $A_{02}$, $A_{10}$, $A_{12}$, $B_{01}$, $G_{04}$; Figure S2.4: The pie charts matrix representing the categorical analysis for drug–drug interactions between drugs in subcategories $C_{07}$, $C_{08}$, $C_{09}$, $C_{10}$, and $M_{01}$, $M_{04}$, $N_{05}$-1, $N_{05}$-2, $N_{06}$-1, $N_{06}$-2.

## Abbreviations

The following abbreviations are used in this manuscript:

DDI	Drug–drug interaction
EDIC	Electronic drug interaction checker
CVD	Cardiovascular disease
ATC	Anatomical Therapeutic Chemical
LDL	Low-density lipoprotein
HDL	High-density lipoprotein
