# Supplementary material for: Categorical Analysis of Database Consistency in Reporting Drug–Drug Interactions for Cardiovascular Diseases
Source: Pharmaceutics. 2024 Feb 28;16(3):339. doi: 10.3390/pharmaceutics16030339 (PMC10975099; doi:10.3390/pharmaceutics16030339)
Supplement: Supplementary file 1 [file pharmaceutics-16-00339-s001.zip › pharmaceutics-2813966-supplementary-update.pdf]

Categorical analysis of database consistency in reporting drug-drug interactions for cardiovascular diseases

Supplementary material

Liana Suciu[1,2†], Sebastian Mihai Ardelean[3,†], Mihai Udrescu[3,*], Florina-Diana Goldiş[4], Daiana Hânda[4], Maria-Medana Tuică[4], Sabina-Oana Vasii[4], and Lucreţia Udrescu[4,*]

[1] Department II—Pharmacology, Pharmacotherapy, "Victor Babeş" University of Medicine and Pharmacy Timişoara, 300041 Timişoara, Romania

[2] Research Center for Pharmaco-Toxicological Evaluations, "Victor Babeş" University of Medicine and Pharmacy Timişoara

[3] Department of Computer and Information Technology, University Politehnica of Timişoara, 300223 Timişoara, Romania

[4] Department I—Drug Analysis, "Victor Babeş" University of Medicine and Pharmacy Timişoara, 300041 Timişoara, Romania

[*] Correspondence to: udrescu.lucretia@umft.ro; mihai.udrescu@cs.upt.ro

[†] These authors contributed equally to this work.

List of Figures

S1.1 The symmetric matrix of pie charts illustrating the categorical analysis for drug-drug interactions between cardiovascular drugs in subcategories C_{01}, C_{02}, C_{03}, and C_{04}. . . . 2

S1.2 The pie charts matrix representing the categorical analysis for drug-drug interactions between cardiovascular drugs in subcategories C_{01}, C_{02}, C_{03}, C_{04}, and C_{07}, C_{08}, C_{09}, C_{10}. 3

S1.3 The symmetric matrix of pie charts illustrating the categorical analysis for drug-drug interactions between cardiovascular drugs in subcategories C_{07}, C_{08}, C_{09}, and C_{04}. . . . 3

S2.1 The pie charts matrix representing the categorical analysis for drug-drug interactions between drugs in subcategories C_{01}, C_{02}, C_{03}, C_{04}, and A_{02}, A_{10}, A_{12}, B_{01}, G_{04}. 4

S2.2 The pie charts matrix representing the categorical analysis for drug-drug interactions between drugs in subcategories C_{01}, C_{02}, C_{03}, C_{04}, and M_{01}, M_{04}, N_{05}-1, N_{05}-2, N_{06}-1, N_{06}-2. 4

S2.3 The pie charts matrix representing the categorical analysis for drug-drug interactions between drugs in subcategories C_{07}, C_{08}, C_{09}, C_{10}, and A_{02}, A_{10}, A_{12}, B_{01}, G_{04}. 5

S2.4 The pie charts matrix representing the categorical analysis for drug-drug interactions between drugs in subcategories C_{07}, C_{08}, C_{09}, C_{10}, and M_{01}, M_{04}, N_{05}-1, N_{05}-2, N_{06}-1, N_{06}-2. 5

We consider DDI between drugs of the cardiovascular category C and DDI between drugs of the cardiovascular category C and others category O. We use the ATC level 2 listed by DrugBank to select drug subcategories included in the Cardiovascular System category C. We include in C all approved drug molecules listed by DrugBank in subcategories C_{01}–*Cardiac therapy*, C_{02}–*Antihypertensives*, C_{03}–*Diuretics*, C_{04}–*Peripheral vasodilators*, C_{07}–*Beta blocking agents*, C_{08}–*Calcium channel blockers*, C_{09}–*Agents acting on the renin-angiotensin system*, and C_{10}–*Lipid modifying agents*. Then, we divide the other drugs in O as non-cardiovascular approved drugs that interact with drugs in C, listed by DrugBank in the level 2 ATC, in the following subcategories: A_{02}–*Drugs for acid-related disorders*, A_{10}–*Drugs used in diabetes*, A_{12}–*Mineral supplements* (only potassium salts), B_{01}–*Antithrombotic agents*, G_{04}–*Urologicals* (only drugs used in the benign prostatic hypertrophy), M_{01}–

Antiinflammatory and antirheumatic products, M_{04}–*Antigout preparations*, N_{05}–*Psycholeptics* splitted into N_{05}-1–Antipsychotics and N_{05}-2–Hypnotics, sedatives, anxiolytics, and N_{06}–*Psychoanaleptics* splitted into N_{06} into N_{06}-1–Antidepressants and N_{06}-2–Nootropics and anti-dementia.

We build two lists that contain drug pairs only between drugs from distinct subcategories in C and drugs in subcategories of C and O, respectively; for all these pairs, we manually check the DDI severity on Drugs.com and WebMD.com. The unified levels of DDI severity in the two drug databases, WebMD.com and Drugs.com, are *No interaction found*, *Minor*, *Moderate*, *Major*, and *Contraindicated*.

We consider an agreement between Drugs.com and WebMD.com if the severity levels indicated by each database coincide. We further check for DDIs included in the Agreement category the distribution of levels of agreement, namely the percentage of DDIs listed in both drug databases as *Not found*, *Minor*, *Moderate*, *Major*, and *Contraindicated*. In contrast, we consider a disagreement between Drugs.com and WebMD.com if they report different results for a tested DDI.

Section 1 presents the categorical analysis of DDI between drugs from the cardiovascular drug subcategories.

Section 2 presents the categorical analysis of DDI between drugs in the cardiovascular and other drug subcategories.

The matrices with pie charts show the corresponding apportionment of DDIs in one of the following categories: Agreement, contraindicated; Agreement, major; Agreement, moderate; Agreement, minor; Agreement, not found; Disagreement. The main diagonals of the symmetric matrices in Figures S1.1 and S1.2 show vertical bars because we do not check DDI between drugs of the same subcategory.

S1 Interactions between cardiovascular drugs: categorical agreement visualization

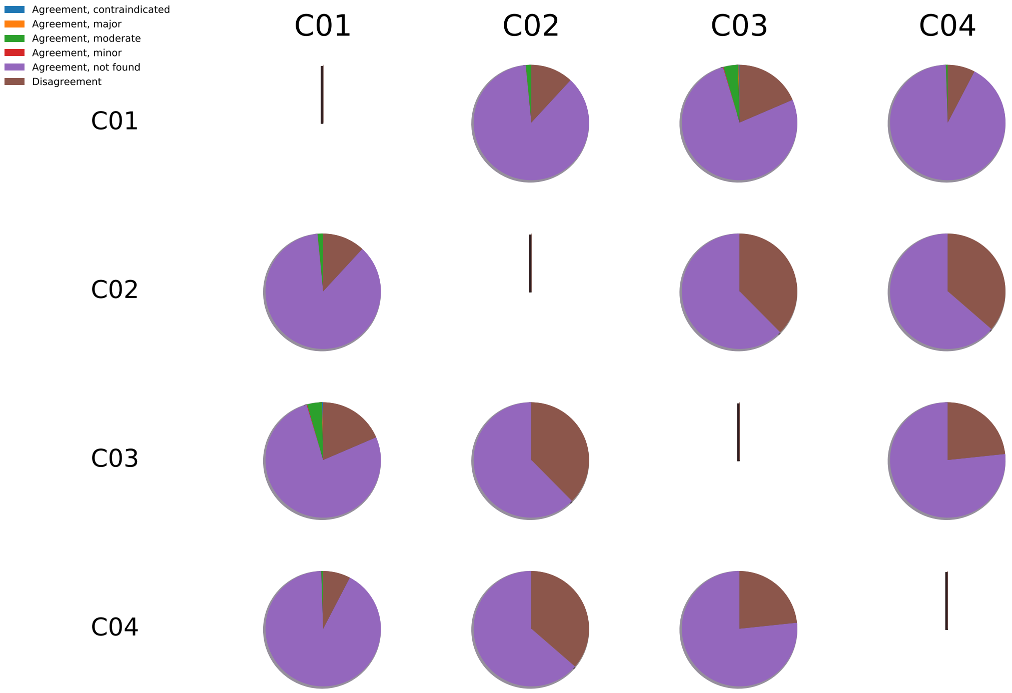

Figure S1.1: The symmetric matrix of pie charts illustrating the categorical analysis for drug-drug interactions between cardiovascular drugs in subcategories C_{01}, C_{02}, C_{03}, and C_{04}.

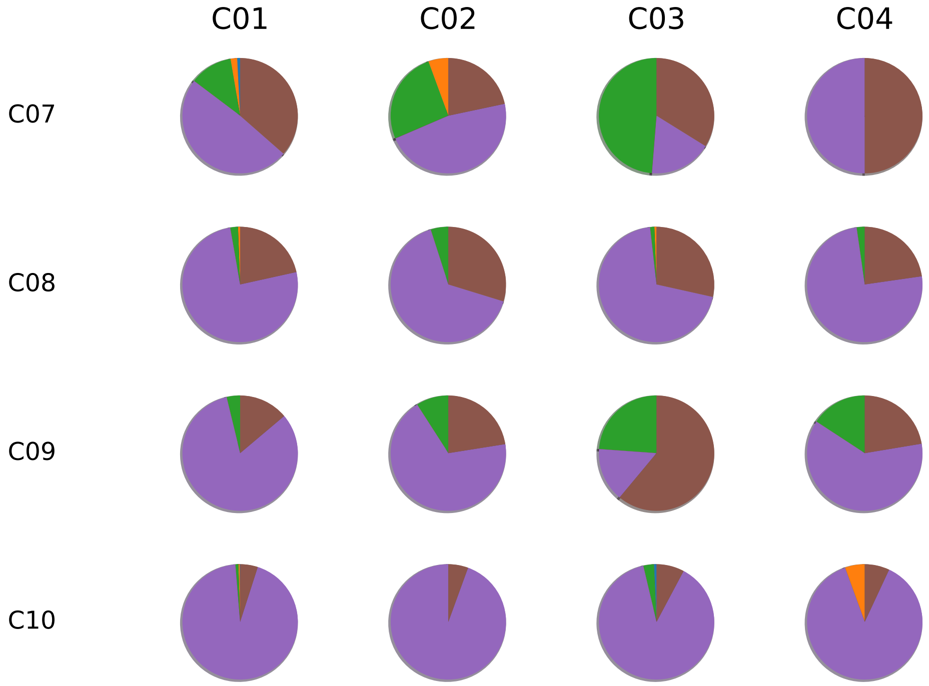

Figure S1.2: The pie charts matrix representing the categorical analysis for drug-drug interactions between cardiovascular drugs in subcategories C_{01}, C_{02}, C_{03}, C_{04}, and C_{07}, C_{08}, C_{09}, C_{10}.

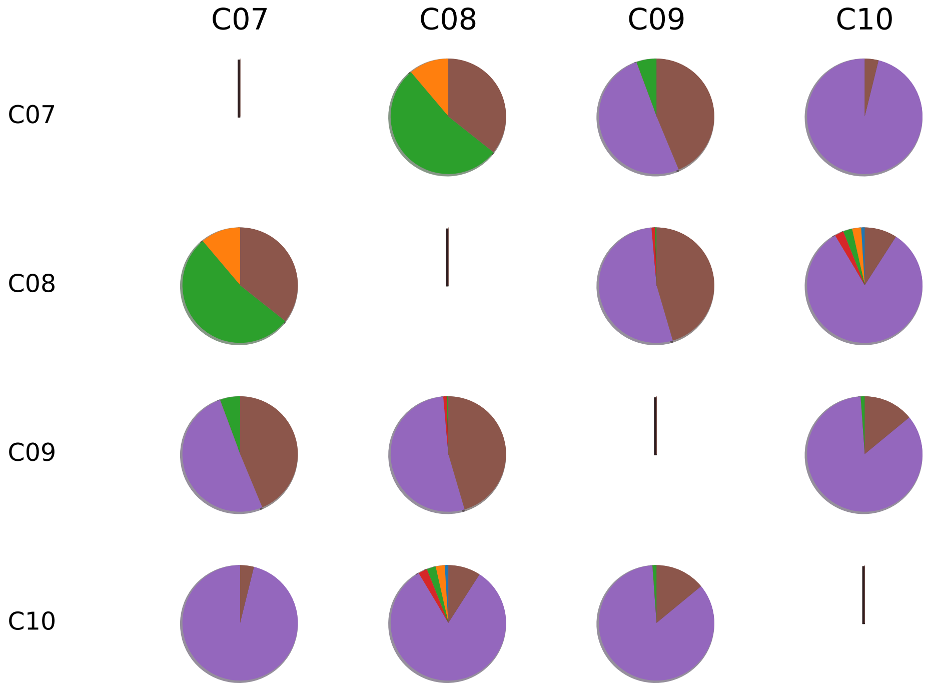

Figure S1.3: The symmetric matrix of pie charts illustrating the categorical analysis for drug-drug interactions between cardiovascular drugs in subcategories C_{07}, C_{08}, C_{09}, and C_{04}.

S2 Interactions between cardiovascular and other drugs: categorical agreement visualization

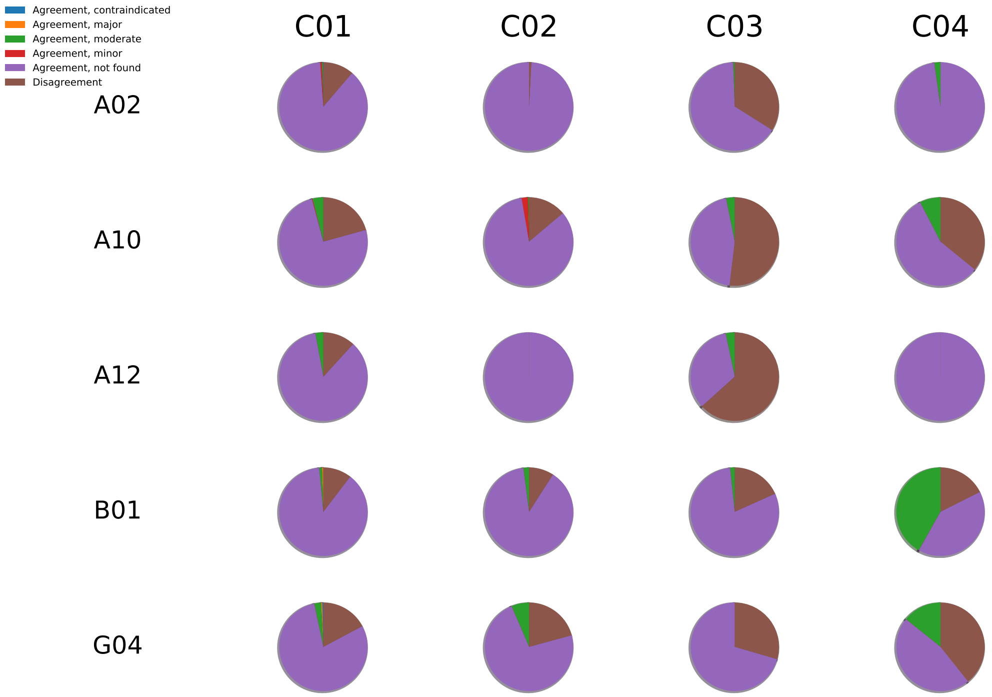

Figure S2.1: The pie charts matrix representing the categorical analysis for drug-drug interactions between drugs in subcategories C_{01}, C_{02}, C_{03}, C_{04}, and A_{02}, A_{10}, A_{12}, B_{01}, G_{04}.

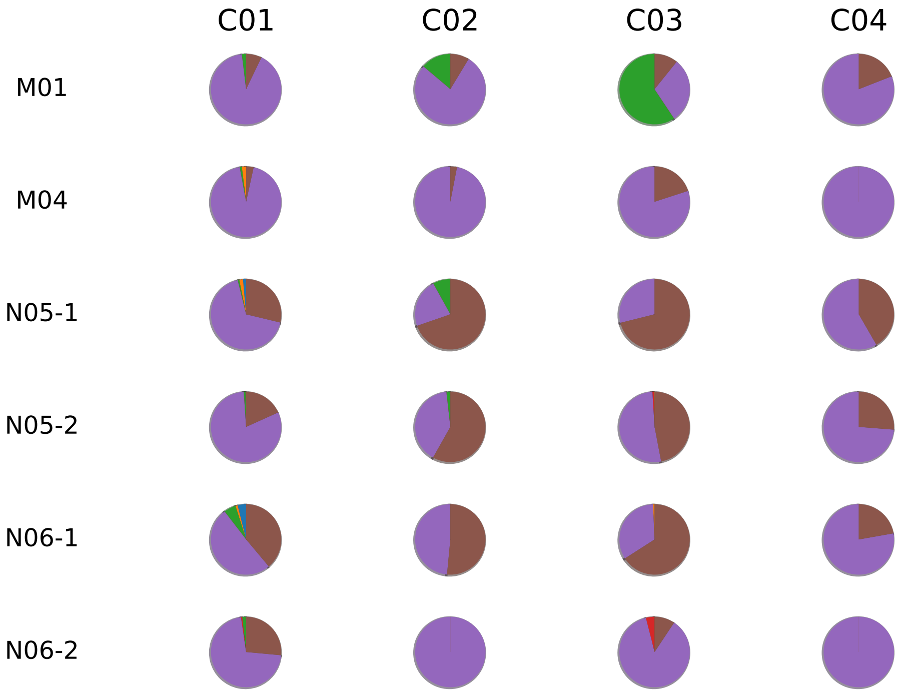

Figure S2.2: The pie charts matrix representing the categorical analysis for drug-drug interactions between drugs in subcategories C_{01}, C_{02}, C_{03}, C_{04}, and M_{01}, M_{04}, N_{05}-1, N_{05}-2, N_{06}-1, N_{06}-2.

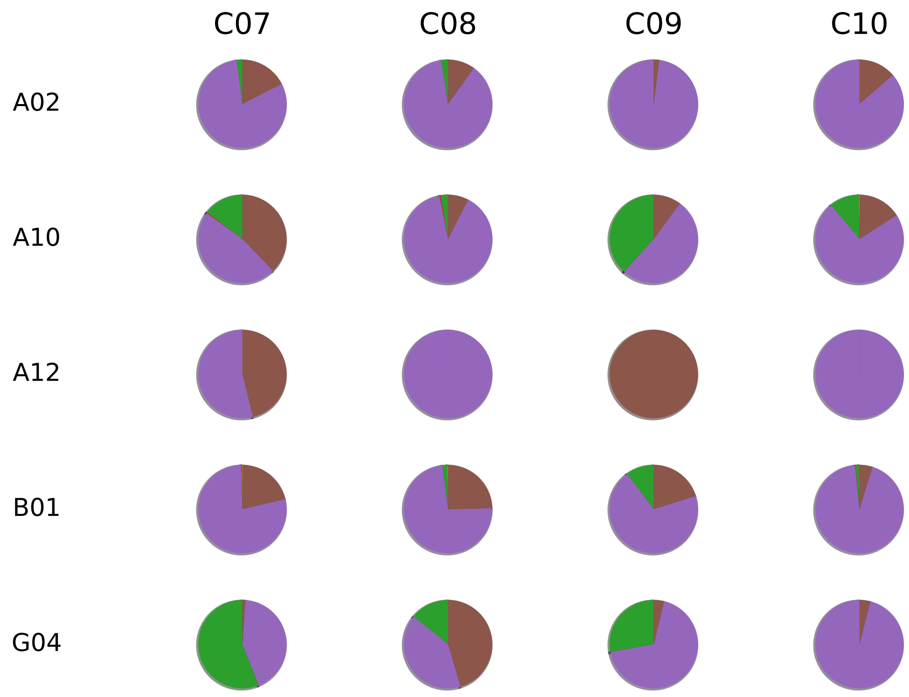

Figure S2.3: The pie charts matrix representing the categorical analysis for drug-drug interactions between drugs in subcategories C_{07}, C_{08}, C_{09}, C_{10}, and A_{02}, A_{10}, A_{12}, B_{01}, G_{04}.

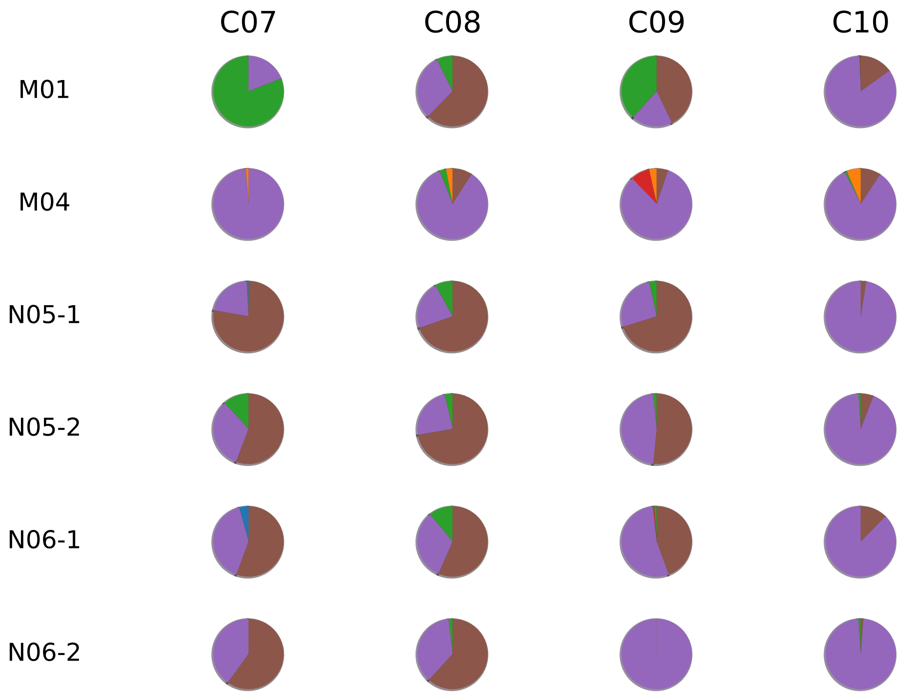

Figure S2.4: The pie charts matrix representing the categorical analysis for drug-drug interactions between drugs in subcategories C_{07}, C_{08}, C_{09}, C_{10}, and M_{01}, M_{04}, N_{05}-1, N_{05}-2, N_{06}-1, N_{06}-2.
